# Adsorption Analyses of Phenol from Aqueous Solutions Using Magadiite Modified with Organo-Functional Groups: Kinetic and Equilibrium Studies

**DOI:** 10.3390/ma12010096

**Published:** 2018-12-28

**Authors:** Mingliang Ge, Xubin Wang, Mingyi Du, Guodong Liang, Guoqing Hu, Jahangir Alam S.M.

**Affiliations:** 1 Key Laboratory of Polymer Processing Engineering of Ministry of Education, National Engineering Research Center of Novel Equipment for Polymer Processing, School of Mechanical & Automotive Engineering, South China University of Technology, Guangzhou 510640, China; gml@scut.edu.cn (M.G.); xubinwang66@163.com (X.W.); dumingyisir@163.com (M.D.); gqhu@scut.edu.cn (G.H.); 2 Key Laboratory of Polymeric Composite & Functional Materials of Ministry of Education, Sun Yat-Sen University, Guangzhou 510275, China; lgdong@mail.sysu.edu.cn; 3 Department of Robotics & Mechatronics Engineering, University of Dhaka, Dhaka 1000, Bangladesh; 4 Department of Computer Science & Engineering, Jessore University of Science and Technology, Jessore Sadar 7408, Bangladesh

**Keywords:** adsorption, phenol, magadiite, adsorption kinetics, adsorption isotherms

## Abstract

Organically-modified magadiite (MAG–CTAB–KH550) was synthesized via ion-exchange method and condensation reaction in the presence of pure magadiite (MAG), cetyltrimethylammonium bromide (CTAB) and γ-aminopropyltriethoxysilane (KH550) in aqueous solution in this research. This new adsorbent material was studied using scanning electron microscope (SEM), X-ray diffraction (XRD), Fourier transforms infrared spectroscopy (FTIR), and N_2_ adsorption/desorption isotherms process. It was found that the MAG–CTAB–KH550 has high Brunaur-Emmet-Teller (BET) specific surface area and mesoporous pore size distribution which enhanced its ability to remove phenol in aqueous solution; and, the value of pH has a relatively large impact on the adsorption behavior of the sorbent. Finally, the adsorptive behavior of the mesoporous material on phenol was followed pseudo-second-order kinetic adsorption model. In contrast, the adsorption equilibrium isotherm was better performed Langmuir isotherm model than the Freundlich isotherm model; in addition, the results also showed that the MAG–CTAB–KH550 had a better adsorption capacity and removal efficiency than MAG.

## 1. Introduction

With the rapid development of the economy and the improvement of people’s living standards, increasingly serious environmental problems become the focus of the whole society, as reported by one researcher [1]. That researcher reported that organic pollution is one of the leading causes of the deterioration of the water environment. Phenolic compounds [2], which are typical organic pollutants, serve as important raw materials for preservatives, fungicides and drugs; however, large amounts of phenol wastewater are generated in the coatings, pesticides, printing, and other industrial fields [3]. Other researchers have shown that, at present, the chemical and biochemical approaches are the most studied water purification methods for eliminating organic pollutants, while poor treatment effects and higher operating costs hinder their extensive development [4,5]; therefore, finding a cheap and efficient method for water purification is imperative and valuable. In contrast, another research investigation reported that adsorption methods could effectively remove a variety of organic and inorganic pollutants from sewerage through three mechanisms, i.e., physical adsorption, chemical adsorption, and ion exchange adsorption [6]. But, after contaminants were removed by the adsorption method using adsorbents, the water had original quality and stability; therefore, the development of new pollutant adsorbents has gradually become a research hotspot [7,8,9]. Various activated carbons and silicate clays serve as the most widely used adsorbents due to their high adsorption capacities for organic pollutants, i.e., phenols and Zn^2+^, by using montmorillonites as sorbents, as described by Wang et al. The adsorption results showed that the organic montmorillonites have much larger adsorption capacities for phenol than pristine montmorillonites [10,11]. However, other adsorbents such as coals [12], Kaolinite [13], pillared clays [14], and so on all shared the same characteristics of larger specific surface areas and porous structures [15]. Scientists have also found that many other porous materials with chelating functional groups which effectively remove heavy metals and organics from sewerage [16,17]. However, it has been found that few sorbents simultaneously have excellent chemical and thermal stabilities during the adsorption process [18].

Researchers reported that the layered silicate is not only rich in natural reserves, but also has a large specific surface area and excellent pollutant adsorption capacity; therefore, it is considered one of the most promising substances for use as an adsorbent [19]. The use of different types and compositions of organic surfactants to modify layered silicates to enhance the adsorption capacity of organic pollutants in water is one of the hotspots in environmental remediation and wastewater treatment [20,21]. Later, researchers reported on a layered silicate of magadiite which has high purity, ion exchange capacity, and possesses good thermal as well as chemical stability [22]. In addition, the layers of magadiite have a large amount of exchangeable water and sodium ions, which determines that they have good ion exchange properties [23]; however, objects ranging from protons [24] to polymers [25] that can be inserted into the layers make the layered magadiite have a proper interlamellar spacing. Therefore, Yoshiaki et al. used reagent octyltrichlorosilane 3-aminopropyltriethoxysilane as the intermedium to be intercalated to magadiite when they prepared a novel pillared magadiite. In this study, they showed that intercalated magadiite had significant improvement interlayer spacing and surface area [26]. Moreover, the composite materials obtained by intercalating the guest molecules onto the inorganic layer have the chemical properties of both inorganics and guest molecules, which indicates a bright future in the adsorption field for magadiite [27]; thus, this intercalation could also render the magadiite surface more hydrophobic or hydrophilic, depending on the nature of the intercalated molecules [28]. Consequently, one of the strategies towards the improvement of the adsorption capacity of magadiite consists of the incorporation of organic functionalities with high affinities for certain organic compounds [29]. He et al. selected four surfactants with different tails to regulate the distance of magadiite layers, then investigated the adsorption performances of these materials by the removal of heavy metals. The materials possessed a high adsorption capacity and different selectivity, especially for Cu(II) and Pb(II) [30]; however, Guerra et al. [31] extended the magadiite layer spacing with *N*-propyldiethylene-trimethoxysilane and bis[3-(triethoxysilyl) propyl]tetrasulfide, and studied the adsorption performance on arsenic(III). Fujita et al. [32] investigated the adsorption performance of 1-hexanol and 1-butanol from water on organic magadiite which had been modified by sialylation with octyltrichlorosilane. Thus, the removal efficiency and selectivity depended on the spacing and equipping of interlayers as well as the types of heavy metal determination. To date, there have also been many adsorption behavior studies of magadiite for other organic pollutants and metal ions. Furthermore, Nunes et al. [33] studied the adsorption performance of magadiite for removing pesticide 2,4-D, diuron and atrazine from water; and, Guerra [34] studied the change in adsorption performance of the 2-mercaptopyrimidine compound attached to the synthesized Na-magadiite sample by the homogeneous route. This research study and its findings can be summarized as follows. (i) The intercalated substance played a dominant role in the maximum uptake capacity for certain organics and metal ions, which provides the possibility of magadiite being applied in adsorption field (ii) The MAG–CTAB–KH550 modified by both cationic surfactant (cetyltriethylammnonium bromide) and coupling agent (γ-Aminopropyltriethoxysilane) was synthesized and characterized by SEM, XRD, and FTIR. Its specific surface area, pore size, and the pore volume were investigated by N_2_ adsorption/desorption isotherms. (iii) Based on the above considerations and the harmfulness of phenolic compounds to the environment, phenol was used as an adsorbate to investigate the adsorption capacity and removal mechanisms of MAG and MAG–CTAB–KH550 under different pH conditions, adsorbent dosages, contact times, etc. (iv) The pseudo-first order and pseudo-second order have been used to fit the kinetic data. (v) The Freundlich and Langmuir adsorption isotherms have been used to perform the model of the equilibrium adsorption data. And finally, (vi) the results of this work have provided useful information for the development of new and effective MAG–CTAB–KH550 adsorbents for the uptake of organic pollutants from sewerage. 

## 2. Experimental

### 2.1. Sorbent Materials Collection

The pristine magadiite used in this study was prepared in a laboratory [35]. The coupling agent, γ-Aminopropyltriethoxysilane (KH550, 99%), was purchased from the Jinan Xingfeinong Chemical Company (Jinan, China). Phenol served as the analytical-grade reagent, which was purchased from Tianjin Damao Chemical Reagent Factory (Tianjin, China). Deionized water was obtained through the laboratory of South China University of Technology, China. Other chemicals of reagent grade were involved in all analytical-grade reagents and purchased from Guangzhou Qianhui Company (Guangzhou, China), except for the cationic surfactant, cetyltrimethylammonium bromide (CTAB, 99%), which was purchased from Tianjin Fuchen Chemical Reagent Factory (Tianjin, China). 

### 2.2. Adsorbent Preparation

**Figure 1:**
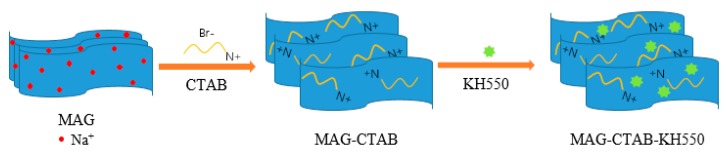
Procedure of MAG–CTAB–KH550 preparation.

MAG–CTAB–KH550 was prepared by using pristine MAG, CTAB, and KH550 as follows: MAG was dispersed in deionized water and placed in ultrasonic conditions for 1 h; a certain amount of CTAB and deionized water was mixed with the well-dispersed suspension of MAG. Then, the mixtures were magnetically stirred for 6 h at 60 °C. The obtained product was washed and filtered with deionized water until no Br^–^ was detected in the filtrate with AgNO_3_ solution. This was followed by drying at 80 °C. The compound (named MAG–CTAB) was then ground for later use. In the next step, a quantity of KH550 was uniformly dissolved in the MAG–CTAB suspension. The ready-prepared solution was vigorously stirred at 60 °C for 6 h. The precipitate was washed with water three times and dried at 80 °C for 12 h to yield the final adsorbent, named MAG–CTAB–KH550. The experimental process is shown in Figure 1.

### 2.3. Adsorption Experiments and Calculations

The effects of the adsorbent dosage, pH value, adsorption time, and initial phenol concentration were investigated in this research study on the adsorption behavior of MAG and MAG–CTAB–KH550 at 25 °C. Before performing the adsorption experiments in this research study, an ultraviolet spectrophotometer (Youke Instrument Company, Shanghai, China) was used to measure the absorption spectrum curve of phenol. It was found that phenol has a significant absorption peak at a wavelength of 270 nm. Therefore, it was observed that after adsorbing by MAG and MAG–CTAB–KH550, the concentrations of phenol solution were centered at 270 nm by testing for absorbance. The influence of the different sorbents dosages on the adsorption of phenol were investigated by controlling a variable method, which may be described as follows: (i) phenol was diluted into the initial concentration of 100 mg/L solutions in a flask containing deionized water; (ii) a certain amount of MAG–CTAB–KH550 (0.25, 0.5, 0.75, 1, 2, 3, 4 g/L) was added to the solution; (iii) the mixture was stirred at a certain speed to begin adsorption processes; (iv) the process was halted after 60 min of adsorption time, and (v) the solution was detached at 8500 rpm in a centrifuge (XiangZhi Centrifuge Company, Changsha, China) for 10 min. The supernatant liquid was measured using a UV spectrophotometer to get the phenol concentration after adsorption. The phenol adsorption capacity *q_t_* (mg/g) and removal efficiency *P* (%) of MAG–CTAB–KH550 was calculated by mass-balanced equation according to the following:

Adsorption Capacity:

(1) $$q_{t}\left(\mathrm{mg}/g\right)=\frac{C_{0}-C_{t}}{m}\times V$$

Removal Efficiency:

(2) $$P\left(\%\right)=\frac{C_{0}-C_{t}}{C_{0}}\times 100\%$$

where *q_t_* (mg/g) was the unit adsorption capacity, *P* (%) was the removal efficiency of phenol, *C*_0_ (mg/L) was the initial concentration of phenol, *C_t_* (mg/L) was the adsorption equilibrium concentration of phenol, *V* was the volume of phenol, *m* was the quality of the sorbents.

The adsorption of phenol by adsorbents with different adsorption times was studied under the condition of an initial concentration of phenol as 50 mg/L and a pH value of 10. In the same processes as those described above, the adsorption times (at 1, 3, 5, 7, 10, 15, 20, 30, 60, 90, 120 min) and the initial concentrations of phenol (at 10, 20, 30, 40, 50, 60, 80, 100 mg/L) were also investigated at the 1 g/L of sorbents dosage. When the influence of pH on the adsorption of phenol had been determined, the initial pH value of the solution was adjusted to within the range of 2–12 with adsorption times of 60 min. Finally, the same experiment as that of the MAG–CTAB–KH550 was performed for MAG in order to compare the adsorption effects.

### 2.4. Characterization Methods

#### 2.4.1. SEM Images

To observe the microscopic morphology changes before and after the intercalation of samples, SEM images were obtained using a field-emission SEM system (Nova Nano SEM 430, FEI, Hillsboro, OR, USA). The operating voltage selection was within the range of 10–20 kV).

#### 2.4.2. FTIR Spectra

The skeleton structure of the sample was measured using an infrared spectrometer (NEXUS Model 670 Fourier, Thermo Nicolet Corporation, Waltham MA, USA). During the scan to subtract the interference of air moisture and carbon dioxide on the sample, the spectral scanning range was 400–4000 cm^−1^, the resolution was set to 2 cm^−1^, and the number of scans was 32.

#### 2.4.3. XRD Analysis

In order to test the change of interlayer spacing before and after intercalation, phase analyses of the samples were performed using XRD (Model D8 ADVANCE, Bruker AXS, Karlsruhe, Germany) with a scan range of 2°–60°, a scan step of 0.02°, and a scan rate of 6°/min. In addition, its metal target was a Cu target; the tube current and voltage were set to 40 mA and 40 KV respectively.

#### 2.4.4. Physical Adsorption/Desorption of N_2_ and Specific Surface Area Determination

The nitrogen adsorption isotherms of the samples were determined using a physical adsorption instrument (Micron ASAP-2020, Micromeritics, Norcross, GA, USA.) at liquid nitrogen temperature (−196 °C). Then, the specific surface areas and pore size distributions were calculated using the Brunaur-Emmet-Teller (BET) equation and Barrett-Joyner-Halenda (BJH) method, respectively.

### 2.5. Adsorption Kinetics Study

In order to investigate and explain the adsorption behavior of MAG and MAG–CTAB–KH550 on phenol, the adsorption kinetics including the pseudo-first-order and pseudo-second-order kinetic models were applied in this research [36]. They described the principle of the effects of various factors on the adsorption rates of MAG and MAG–CTAB–KH550.

#### 2.5.1. Pseudo-First-Order Kinetic Model

Based on the assumption of diffusion step control, the pseudo-first-order kinetic equation is shown [37] as

(3) $$\frac{dq_{t}}{dt}=K_{1}\left(q_{e}-q_{t}\right)$$

where *K*_1_ (mg/g min) was the rate constant of the pseudo-first-order adsorption, *q_e_* (mg/g) was the adsorption capacity at equilibrium, and *q_t_* (mg/g) was the adsorption capacity at time *t*. If *q* = 0 at *t* = 0, then

(4) $$ln\left(q_{e}-q_{t}\right)=lnq_{e}-K_{1}t$$

In plotting the value of *ln(q_e_ − q_t_)* vs time *t*, straight lines were obtained. If the kinetic adsorption follows pseudo-first-order kinetics, then the values of *K*_1_ were calculated from the slope of the plots.

#### 2.5.2. Pseudo-Second-Order Kinetic Model

The pseudo-second-order kinetic model was determined by the chemical adsorption mechanism, and it was assumed that the adsorption rate depended on the square of the active adsorptive sites number in the surface of adsorbent. The equation of the model is as follows [38],

(5) $$\frac{t}{q_{t}}=\frac{1}{K_{2}q_{e}^{2}}+\frac{1}{q_{e}}t$$

where *K*_2_ (g/mg min) is the constant of the pseudo-second-order rate. *q_t_* and *q_e_* were the same as depicted above for the pseudo-first-order kinetics model.

### 2.6. Adsorption Isotherms

Adsorption isotherms describe the theoretical adsorption maximum capacity of quantitative adsorbents, and determined the feasibility of adsorbent dosage; however, they serve as important reference values in the design process of the adsorption system. In this study, the adsorption isotherms were plotted using the Langmuir and Freundlich adsorption isotherms [39,40].

#### 2.6.1. Langmuir Isotherm Model

The theoretical basis of the Langmuir isotherm model states that the adsorbed molecules occupy the finite adsorption sites of homogenous surface by monolayer adsorption, and that there is no interaction between adsorbed molecules in adsorption process [39]. The empirical equation is as follows:

(6) $$\frac{C_{e}}{q_{e}}=\frac{1}{K_{L}q_{m}}+\frac{C_{e}}{q_{m}}$$

where *q_e_* (mg/g) is the amount of phenol adsorbed per gram of sorbent at equilibrium, *q_m_* (mg/g) is the maximum amount of adsorption, *K_L_* (L/mg) is the Langmuir equilibrium constant related to the affinity of binding sites and energy of adsorption, and *C_e_* (mg/L) is the equilibrium concentration of substrates in the solution. Values of *K_L_* and *q_m_* were calculated by the intercept and slope of the liner plot of experimental dates of *C*_o_/*q_e_* versus *C_e_*. A dimensionless constant of the Langmuir equation called the equilibrium parameter *R_L_* was defined as follows:

(7) $$R_{L}=\frac{1}{1+C_{0}K_{L}}$$

where *C*_0_ (mg/L) was the initial concentration, the value of *R_L_* indicated the type of isotherm to be irreversible adsorption (*R_L_* = 0), favorable adsorption (0 < *R_L_* < 1), unfavorable adsorption (*R_L_* > 1) and linear adsorption (*R_L_* = 1). The closer to zero, the more favorable the adsorption.

#### 2.6.2. Freundlich Isotherm Model

For the Freundlich isotherm, that mainly describes the adsorption equilibrium of multi-phase adsorption surface and which has been extensively employed in developing the model of the adsorption on heterogeneous surface [40], the empirical equation is generally expressed as follows:

(8) $$q_{e}=K_{F}C_{e}{}^{\frac{1}{n}}$$

After integration, equations can also be written as:

(9) $$\ln q_{e}=\frac{1}{n}\ln C_{e}+\ln K_{F}$$

where *q_e_* (mg/g) is the equilibrium adsorption capacity, *C_e_* (mg/L) is the equilibrium concentration of the adsorbate, *K_F_* is the Freundlich constant representing the adsorption capacity, and *n* is a constant depicting the adsorption intensity.

## 3. Results and Discussions

### 3.1. Characterization of Adsorbents

**Figure 2:**
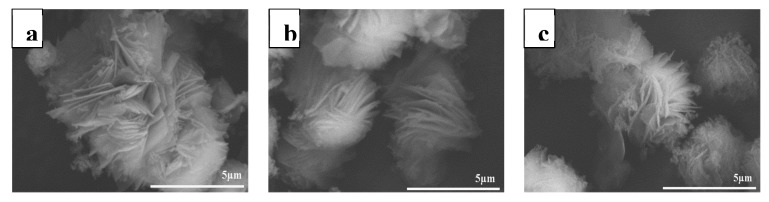
SEM images of (**a**) MAG; (**b**) MAG–CTAB and (**c**) MAG–CTAB–KH550.

The shape of the MAG was a layered structure of rosette-like petals [41], as shown in Figure 2a. The crystal morphology was uniform; the size of the entire spherical petal was approximately 5 μm. The MAG was modified by CTAB and KH550, and the interlayer spacing of MAG–CTAB and MAG–CTAB–KH550 was increased significantly relative to MAG, as shown in Figure 2b,c. CTAB and KH550 not only covered the surface and edges of MAG, but also inserted into the MAG layer space. The crystal morphology and the size of the MAG–CTAB–KH550 were no longer uniform but the rosette-like petals were stretched all around. The phenomenon described the originally-modified process. Two modification mechanisms were performed as ion-exchange reactions between quaternary ammonium cation and Na^+^ occurred in the MAG gallery, and the long alkyl branch of quaternary ammonium cation helped increase the interlayer spacing [24]. On the other hand, the chemical reaction occurred in the time when MAG was modified by KH550; and KH550 was easy to hydrolysize. It then lost three ethoxy groups to form covalent bonds with the silicon hydroxyl on the surface of MAG, as reported previously [26].

**Figure 3:**
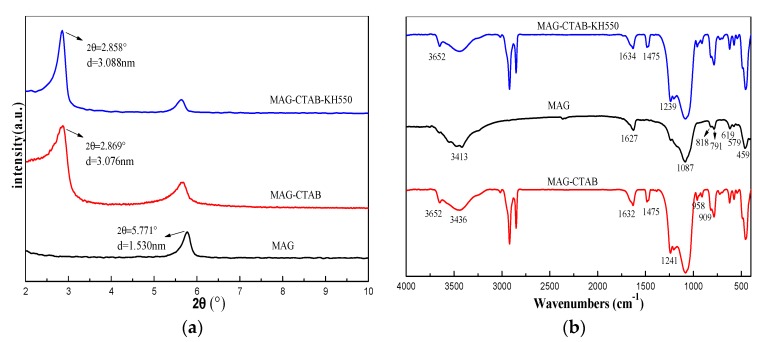
(**a**) XRD patterns of MAG, MAG–CTAB, and MAG–CTAB–KH550; (**b**) Infrared spectrum of MAG, MAG–CTAB, and MAG–CTAB–KH550.

Figure 3a shows the XRD patterns of MAG–CTAB–KH550 compared with the MAG and MAG–CTAB. It was found that the characteristic peaks of MAG were observed at 2θ = 5.77°, corresponding to a 001-plane basal spacing (*d*_001_) of 1.53 nm as reported in [23]. After intercalation with CTAB, the cation exchange between cetyltriethylammnonium cation and Na^+^ brought about the expansion of magadiite’s interlayer spacing. From the patterns, a new diffraction peak appeared at 2θ = 2.869°, and the basal spacing expanded to 3.08 nm via calculation because of the long chain of cetyltriethylammnonium [22]. This indicates the successful interaction of surfactant cation in the layer of MAG. However, after KH550 intercalated into the MAG–CTAB, the intensity of the *d*_001_ peak was strengthened and shifted slightly to a smaller degree, with interlayer spacing of 3.09 nm [42]. This mainly indicated that KH550 has a small effect on the interlayer spacing of MAG–CTAB.

Figure 3b shows the infrared spectrum of MAG, MAG–CTAB, MAG–CTAB–KH550. As seen from the curve of MAG, the asymmetric stretching vibrational absorption peaks of SiO_4_ tetrahedron in magadiite appeared at 1087 cm^−1^, and symmetric stretching vibrational absorption peaks of Si–O appeared at 791 cm^−1^, 459 cm^−1^. The absorption peaks at 3413 cm^−1^ was attributed to the stretching vibrations the hydroxyl group of the MAG layer structure; another bending vibration peak of H_2_O appeared at 1627 cm^−1^ [41].

Compared with the infrared spectrum of MAG, some new characteristic absorption peaks in the curve of MAG–KH550 and MAG–CTAB–KH550 were found as 2925 cm^−1^, 2854 cm^−1^, and 1475 cm^−1^, which were attributed to the asymmetrical, symmetric stretching, and bending vibrational absorption of –CH_2_–. The peak at 1241 cm^−1^ belonged to the characteristic absorption peaks of the stretching vibration of C–N. In the modification process, the intensity of adsorption peaks of Si–OH and Si–O–Si changed slightly, and the former decreased but the latter reversed. This situation indicated that the KH550 produced chemical reactions between its functional group ethoxy and the Si–OH of MAG surface to form Si–O–Si bonds. Therefore, it may be noted that these newly-emerging absorption peaks proved that CTAB and KH550 performed the intercalation into magadiite interlayer by ion-exchange method and condensation reaction. 

### 3.2. The Specific Surface Areas and Pore Size Distributions Calculations

**Figure 4:**
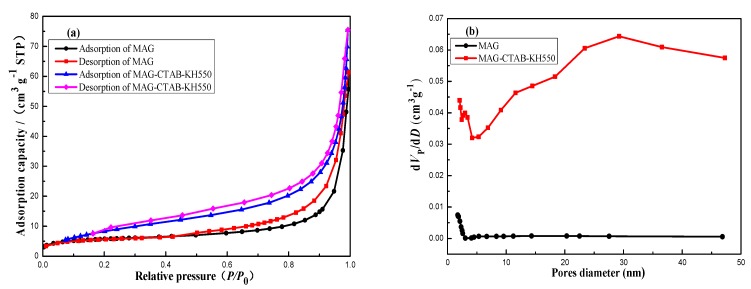
N_2_ adsorption/desorption (**a**) curve and pore size distribution and (**b**) of MAG and MAG–CTAB–KH550 (at 10 s of equilibration interval, and −196 °C of analysis bath temperature).

A N_2_ adsorption/desorption isotherm was carried out for the MAG and MAG–CTAB–KH550 samples in order to evaluate the permanent porosity, specific surface areas, and pore diameter distribution. As shown in Figure 4a,b, two isotherms were slightly bent in the low-pressure region (relative pressure of *P*/*P*_0_ < 0.05) because of superimposition between the monolayer coverage and the initial amount of multi-layer adsorption. Compared with MAG–CTAB–KH550, the MAG demonstrated a horizontal trend at relative pressure in the range of 0.05 < *P*/*P*_0_ < 0.5, which increased sharply after the relative pressure was increased to the range of *P*/*P*_0_ > 0.8. The weak interaction between MAG and nitrogen has been reported [10]. However, modifier agents enhanced the adsorptive interaction; thus, MAG–CTAB–KH550 exhibited earlier upward tendencies of adsorption capacity. According to these traits, it could be considered that the N_2_ adsorption/desorption of the two materials is type II isotherm. On the other hand, the adsorption/desorption isotherms followed the hysteresis loop of H3 type in International Union of Pure and Applied Chemistry (IUPAC) classification [43]. This indicates that the MAG and MAG–CTAB–KH550 consisted of slit hole and mesoporous structures formed by stacking of flaky particles [44]. Analysis was performed in accordance with conclusion for MAG’s morphology analysis. In addition, the hysteresis loop starting point of MAG was performed behind MAG–CTAB–KH550; it indicated that more mesoporous structures exist in MAG. The data of adsorption from 0.05–0.35 of *P*/*P*_0_ was applied to determine the specific surface area, mesoporous volume, pore diameter, sample mass, and constant value, by the BET equation and BJH method as well as the Nitrogen adsorption volume by standard temperature and pressure (STP); details are given in Table 1. After intercalating MAG by CTAB and KH550, it was found that the specific surface area and pore volume increased by 58%, and 54%, respectively. Figure 4b illustrates that MAG-CTAB–KH550 has wider pore distribution, which is one of the characteristics of mesoporous materials. These results had very obvious effects which may be because CTAB played a pillared-reagent role in the MAG interaction to increase the adsorption space [11], and because the KH550 modified the surface of the MAG sheet to raise the number of adsorption positions.

### 3.3. Effects of Factors on the Phenol Adsorption

#### 3.3.1. Effects of Adsorbent Dosage

**Figure 5:**
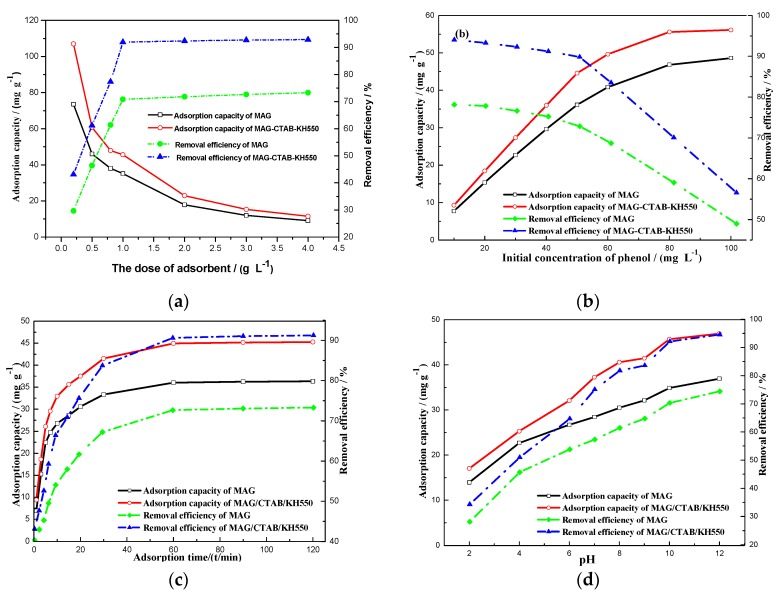
(**a**) Effects of adsorbent dosage of MAG and MAG–CTAB–KH550 on removal efficiency and adsorption capacity of phenol (in the conditions of pH = 10, and the adsorption time for 60 min); (**b**) Effects of initial concentration of phenol on the removal efficiency and adsorption capacity under the conditions of sorbents dosage of 1 g/L and adsorption equilibrium time for 60 min, and pH value of 10; (**c**) Effects of adsorption time on adsorption capacity of phenol under the conditions of initial concentration of phenol at 50 mg/L, pH of 10, and the sorbents dosage of 1 g/L; and (**d**) Effects of pH values of the solution on adsorption capacity of phenol under the conditions of initial concentration of phenol at 50 mg/L, sorbents dosage of 1 g/L, and adsorption equilibrium time of 60 min.

As shown in Figure 5a, the appropriate adsorbent dosage can lessen sewage treatment costs. With increasing the dosage of adsorbents from 0.25 to 4 g/L, the removal efficiency of phenol also increased from 29.66% to 73.31% and 43.13% to 92.88% for MAG and MAG–CTAB–KH550, respectively. As a whole, the adsorption capacity declined gradually. It was clearly shown that the removal efficiency of sorbents increased rapidly, as expected when the dose of sorbents was under 1 g/L. Comparing the MAG before and after modification, the removal efficiency of sorbents of MAG–CTAB–KH550 (after modification) increased by about 45% when the amount of adsorbent was 0.25 g/L, but it increased by 27% at 1 g/L; thus, it was confirmed without doubt that its adsorption capacity was better than that of MAG (before modification). According to the microstructures of sorbents, the reason for the enhancement of the removal efficiency could be attributed to the expansion of slice gap of MAG that provided even more physical adsorption active sites for adsorbates [29]. Nevertheless, there was almost no fluctuation in the removal efficiency behind 1 g/L of adsorbents dose in the curve, because it was shown that stacks occurred among the MAG nanosheets during the adsorption process with increasing concentrations of sorbents, which resulted in active physics adsorption point overlaps and effective adsorption reductions [31]. Therefore, the ideal dose of adsorbents was found to be 1 g/L for both MAG and MAG–CTAB–KH550 in this research study.

#### 3.3.2. Effects of Initial Concentration of Phenol

Figure 5b shows the effects of the initial concentration of phenol in the solutions on the removal efficiency and adsorption capacity. The condition(s) of experiments were performed with sorbent dosages of 1 g/L, an adsorption equilibrium time of 60 min, and a pH value of 10. As shown in Figure 5b, the adsorption capacities of MAG and MAG–CTAB–KH550 increased in the ranges of 7.72–48.59 mg/g, and 9.29–56.13 mg/g, respectively. It was shown that the phenol concentration had a non-negligible effect on adsorption capacity [7,11]. The adsorption capacity of the modified MAG (MAG–CTAB–KH550) increased by 15% compared to that of MAG when the initial phenol concentration was 100 mg/L; thus, the organic modification process for MAG improved its adsorption capacity. Although the removal efficiency declined slightly at first, it started to drop with at an almost fixed speed when the initial phenol concentration was over 50 mg/L; however, the initial phenol concentration has a limited influence on adsorbents because the adsorbents reached a saturation state at 1 g/L of adsorbents dosage. The adsorption capacities of similar adsorption materials are shown in Table 2. It may be concluded that MAG and MAG–CTAB–KH550 have certain advantages for phenol adsorption. Furthermore, considering the experiment cost and efficiency in this study, the chosen optimal initial concentration of phenol was 50 mg/L.

#### 3.3.3. Effects of Adsorption Time

Adsorption equilibrium time is an important parameter for designing sewage treatment schemes. The effects of adsorption time ranges from 0–120 min on the adsorption capacity and removal efficiency of sorbents are described in Figure 5c. It is obvious that the adsorption capacity of two adsorbents for phenol go up by increasing the adsorption time at the beginning period. Specifically, under the conditions of initial phenol concentration 50 mg/L, sorbents dosage at 1 g/L and pH value at 10, the adsorption capacity of the sorbents increased quickly within 30 min, especially in the preiod from 0 to 15 min. The researchers found that the adsorption rates responded quickly and it were relatively closed, and that the sorption layer spacing and active sites on adsorbents surface were easily accessible for phenol [16]. Another study found that as the concentration of phenol increased in the MAG interlayer spacing that resulted in the decrease of active sites and layer spacing, the residue phenol took more time to adhere to the free spacing and active sites [18]. It can be noted that MAG has the same equilibrium time as MAG–CTAB–KH550 from the curves due to the fact that it reached the highest value as 36.24 mg/g for MAG, and 45.08 mg/g for MAG–CTAB–KH550 when contact time reached 60 min. This illustrates that the modifying agent has an impact on the adsorption capacity; in the later adsorbent stages, the remaining adsorption capacity of MAG and MAG–CTAB–KH550 was almost unchanged. This reference served to utilize the adsorbents to purify sewage effectively.

#### 3.3.4. Effects of pH Value and Adsorption Mechanisms

The pH value of adsorbate solutions also have an effect on the adsorption process [46]. Figure 5d shows the effect of a pH value in the range of 2–12 on the adsorption capacity and removal efficiency of MAG and MAG–CTAB–KH550. As seen from Figure 5d, increasing the value of pH, the adsorption capacity of MAG and MAG–CTAB–KH550 increased from 41.83–78.44 mg/g, and 46.94–94.63 mg/g, respectively. Obviously, it was concluded that the adsorption capacity of MAG–CTAB–KH550 was better than that of MAG. However, when the was pH > 7, the adsorption capacity and removal efficiency also increased. The adsorption mechanism of MAG and MAG–CTAB–KH550 for phenol on different pH value was related to the ionization of phenol [10] and ion-exchange reaction of MAG in aqueous solution. Firstly, the ionization constant of phenol was *K*_a_ = 10^−9.98^ at 25 °C; and when the pH of the solution was less than 10, the phenol was mainly in the form of a C_6_H_5_OH neutral molecule, because the MAG–CTAB–KH550 had larger interlayer spacing and mesoporous and slit structures, which created lots of active adsorption points for phenol [12]. Thus, the adsorption process predominantly depended on adsorption at the stage of pH < 10. In contrast, when the pH value was more than 10, the phenol was mainly in the form of C_6_H_5_O^–^ anions. Except for physical adsorption, there were multiple forces between the CTAB, KH550, MAG plate and C_6_H_5_O^–^ anions which increased adsorption capacity. To be specific, the quaternary ammonium of CTAB as functional group with a positive charge formed ionic bonds with C_6_H_5_O^–^ anions which neutralized the electrostatic repulsive-force from plates of MAG, and the long chain of CTAB also multiplied the active adsorption positions, in agreement with previous literature [22]. In addition, the functional group -NH_2_ in the molecular structure of KH550 inclined to form hydrogen bonds with phenol anions in alkaline conditions by the Van-der-Waals Force [47]. Therefore, there were also competitive adsorptive reactions between phenol anionics with hydroxyls; and the adsorption behavior of MAG and MAG–CTAB–KH550 on the anionic form of phenol was better than that of phenol in the molecular form, which accords with the trend of the curve shown in Figure 5d.

### 3.4. Study of Adsorption Kinetics

**Figure 6:**
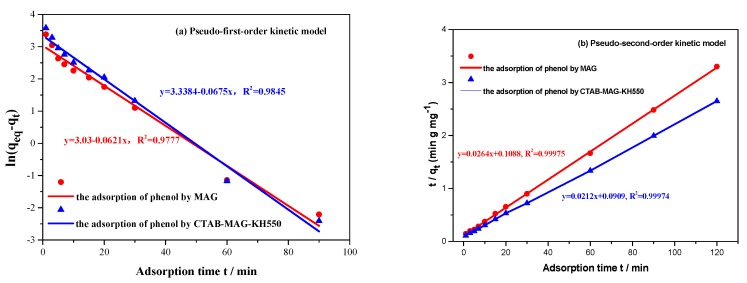
Kinetic curves of the sorption of phenol for pristine MAG and MAG–CTAB–KH550 in the conditions of initial phenol concentration at 50 mg/L, the pH value at 10, and sorbents dosage at 1 g/L for: (**a**) Pseudo-first-order kinetic model; (**b**) Pseudo-second-order kinetic model.

The study of adsorption kinetics study is important to understand the behavior of adsorbate adsorption on various adsorbents, and to contribute to economical adsorption technology. To model phenol adsorption on MAG and MAG–CTAB–KH550, the plots of the pseudo-first-order and pseudo-second-order kinetic model are shown in Figure 6a,b, respectively. The other parameters of both kinetic models were calculated and are presented in Table 3. 

Compared using a correlation coefficient (*R*^2^), the phenol was increased by MAG and MAG–CTAB–KH550, that was well described by the pseudo-second-order kinetic model because its correlation coefficient (*R*^2^) is closed to the value of unity. Based on pseudo-second-order kinetic model, the *q_e_* value order of MAG–CTAB–KH550 (*q_e_* = 37.66 mg·g^−1^) was found to be in front of MAG (*q_e_* = 47.11 mg·g^−1^), which was approximately in line with the *q_et_* value tested by experiments (36.35, 45.26 mg·g^−1^, respectively). It was also suggested that the pseudo-second-order kinetic model showed strongly agreement between the model predicted results and experimental data. On the basis of the pseudo-second-order kinetic model, it was considered that the adsorption process of MAG and MAG–CTAB–KH550 had been controlled by chemical mechanisms [47]. This is consistent with previous adsorption mechanism analyses.

### 3.5. Study of Adsorption Isotherms

**Figure 7:**
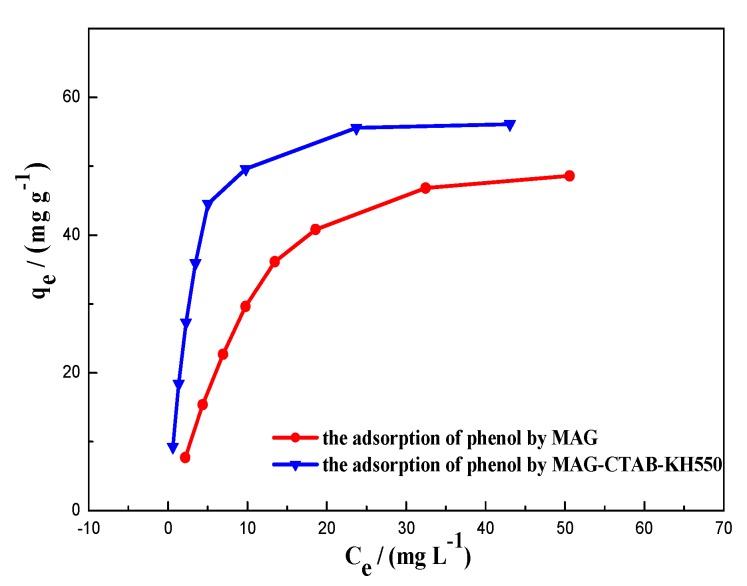
Equilibrium isotherms of adsorption of MAG, and MAG–CTAB–KH550 for phenol.

Adsorption isotherms have certain reference values for explaining the adsorption mechanisms [27]. Figure 7 illustrates the equilibrium isotherms of adsorption of MAG and MAG–CTAB–KH550 on phenol; this describes the specific relationship between the adsorption capacity of adsorbents and the solution concentration of phenol at 25 °C when the adsorption process reached equilibrium at different initial concentrations from 10 to 80 mg/L. As seen in the figure, the adsorption capacity of MAG–CTAB–KH550 increases more rapidly than MAG by increasing the initial concentration of phenol in the early period. This was also confirmed in the previous conclusion that phenol was more quickly adsorbed by MAG–CTAB–KH550. It also could be speculated that something had moderated the interaction between phenol molecules and adsorbents from an ‘L’-shaped curve [48].

**Figure 8:**
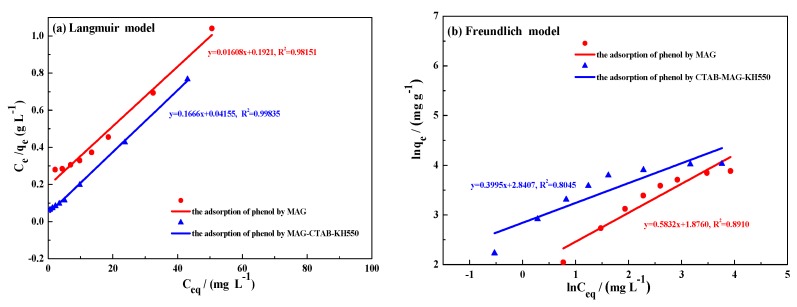
Adsorption isotherm models (**a**) Langmuir model and (**b**) Freundlich model.

The applicability of the Langmuir isotherm model and Freundlich isotherm model in this adsorption experimental data is described as shown in Figure 8a,b. The adsorption parameters of phenol on MAG and MAG–CTAB–KH550 were obtained by the Langmuir and Freundlich models, which are listed in Table 4. Based on these data, it can be noted that, as compared with correlation coefficient (*R*^2^), the Langmuir model exhibited a better fit (*R*^2^ = 0.982 for MAG, and *R*^2^ = 0.998 for MAG–CTAB–KH550) compared to the Freundlich model (*R*^2^ = 0.891 for MAG and *R*^2^ = 0.804 for MAG–CTAB–KH550). Therefore, the adsorption of phenol onto MAG had homogeneous monolayer adsorptions [45]. However, the adsorption favorability through the value of *K_L_* and *n* in Equations (7) and (9) was also determined. In short, these results were consistent with the model analysis that the adsorption process was mainly derived by chemical mechanism [49].

## 4. Conclusions

In this research study, the novel mesoporous material MAG–CTAB–KH550 was prepared by modifying MAG with ion exchange method and condensation reaction. In order to explore the adsorption properties of MAG and MAG–CTAB–KH550 in an aqueous medium, phenol was used as a pollutant. The MAG–CTAB–KH550 has higher BET specific surface area and more even mesoporous pore size distribution compared to MAG, which is one of the reasons why the former has a higher adsorption capacity and stronger removal efficiency. The adsorption capacity and removal rate of the adsorbents were also studied under different conditions. The initial concentration of phenol had the most obviously influence on the adsorption capacity of MAG and MAG–CTAB–KH550, followed by the pH value. Other factors such as sorbent dosage and adsorption time were also investigated in this research study. According to the experimental results, the maximum adsorption capacity of MAG–CTAB–KH550 was 45.26 mg/g in an alkaline environment under an adsorbent dose of 1 g/L, adsorption equilibrium time for 60 min, and an initial concentration of phenol of 50 mg/L. This evidence served as a reference for the effective use of the adsorbent. The adsorption kinetics were found to follow a pseudo-second-order kinetics model to certify the adsorption mechanisms and conform to the chemical adsorption process on both MAG and MAG–CTAB–KH550; however, the physical adsorption process also played an important role in acid or neutral aqueous solutions due to the slit and mesoporous structure of MAG and MAG–CTAB–KH550. In addition, the equilibrium isotherms were better fitted using the Langmuir isotherm than the Freundlich isotherm, which again appeared to illustrate the chemical adsorption mechanisms and mainly explain the phenol adsorption by both MAG and MAG–CTAB–KH550. Furthermore, as the mineral silicate of MAG is environmentally friendly and not poisonous to the human body, it may have potential for use in environmental pollution management.

## Figures and Tables

**Table 1:** Properties of MAG and MAG–CTAB–KH550 by N_2_ adsorption/desorption isotherms analyses.

Isotherms parameters	Adsorbents	
	MAG	MAG–CTAB–KH550
specific surface area (m^2^/g)	21.4174	33.8239
pore volume (cm^3^/g)	0.0756	0.1167
pore size (nm)	14.1259	13.8004
sample mass (g)	0.0751	0.0649
Constant, *C* (BET)	12.9096	22.8386
*V*_m_ (STP)	4.9199	7.7699

**Table 2:** Phenol adsorption on different absorbents at 25 °C with an initial concentration of phenol at 100 mg/L.

Adsorbents	*q_m_* (mg/g)	References
Na-Mt	8.29	[11]
Bentonite	8.435	[13]
Kaolin	2.351	[13]
Zeolite	32.6	[45]
MAG	48.59	This study
MAG–CTAB–KH550	56.13	This study
Active carbon	108.2	[12]

**Table 3:** Adsorption kinetic constants of phenol for MAG and MAG–CTAB–KH550 (pH = 10, 1 g/L of sorbents dosage).

Model	Parameter	Adsorbents	
		MAG	MAG–CTAB–KH550
Pseudo-first-order	*K*_1_ (g·mg^−1^·min^−1^)	0.05916	0.06544
	*q_e_* (mg·g^−1^)	17.34	25.01
	*R* ^2^	0.9777	0.9845
Pseudo-second-order	*K*_2_ (g·mg^−1^·min^−1^)	0.00071	0.00045
	*q_e_* (mg·g^−1^)	37.66	47.11
	*R* ^2^	0.9994	0.9995
Experiments	*q_et_* (mg·g^−1^)	36.35	45.26

**Table 4:** Parameters of isotherm models of phenol adsorption by two adsorbents.

Model	Parameter	Adsorbents	
		MAG	MAG–CTAB–KH550
Langmuir model	*q_m_* (mg/g)	52.19	60.02
	*K_L_* (L/mg)	0.084	0.401
	*R* ^2^	0.982	0.998
Freundlich model	*n*	1.715	2.503
	*K_F_*	6.527	17.128
	*R* ^2^	0.891	0.804
